# Differential contributions of circadian clock genes to cell survival in bipolar disorder patient derived neuronal progenitor cells distinguishes lithium responders and non-responders

**DOI:** 10.21203/rs.3.rs-4331810/v1

**Published:** 2024-04-30

**Authors:** Himanshu K. Mishra, Heather Wei, Melissa LeRoux, Insu Ko, Kayla E. Rohr, Caroline M Nievergelt, Adam X Maihofer, Paul Shilling, Martin Alda, Wade H Berrettini, Joseph R. Calabrese, William H. Coryell, Mark Frye, Elliot Gershon, Melvin G. McInnis, John Nurnberger, Ketil J. Oedegaard, Peter P. Zandi, John R. Kelsoe, Michael J. McCarthy

**Affiliations:** UC San Diego; UC San Diego and VA San Diego; UC San Diego; UC San Diego; UC San Diego; UC San Diego; UC San Diego; UC San Diego; Dalhousie University; University of Pennsylvania; Case Western Reserve University; University of Iowa; Mayo Clinic; University of Chicago; University of Michigan; Indiana University; University of Bergen and Haukeland University Hospital; Johns Hopkins University; UC San Diego; VA San Diego & UC San Diego

**Keywords:** bipolar disorder, stem cell, neuronal progenitor cell, circadian rhythm, apoptosis, lithium

## Abstract

Bipolar disorder (BD) is characterized by disrupted circadian rhythms and neuronal loss. Lithium is neuroprotective and used to treat BD, but outcomes are variable. Past research identified that circadian rhythms in BD patient neurons are associated with lithium response (Li-R) or non-response (Li-NR). However, the underlying cellular mechanisms remain unknown. To study interactions among circadian clock genes and cell survival, and their role in BD and predicting lithium response, we tested selected genes (*PER1*, *BMAL1* and *REV-ERBα*) and small molecule modulators of ROR/REV-ERB nuclear receptors in models of cell survival using mouse neurons and stem-cell derived neuronal progenitor cells (NPC) from BD patients and controls. In apoptosis assays using staurosporine (STS), lithium was neuroprotective. Knockdown of *PER1*, *BMAL1* and *REV-ERBα* modified cell survival across models. In NPCs, reduced expression of *PER1* and *BMAL1* led to more extensive cell death in Li-NR vs. Li-R. Reduced *REV-ERBα* expression caused more extensive cell death in BD vs. control NPCs, without distinguishing Li-R and Li-NR. In IMHN, The REV-ERB agonist GSK4112 had strong effects on circadian rhythm amplitude, and was neuroprotective in mouse neurons and control NPCs, but not in BD NPCs. Expression of cell survival genes following STS and GSK4112 treatments revealed BD-associated, and Li-R associated differences in expression profiles. We conclude that the neuroprotective response to lithium is similar in NPCs from Li-R and Li-NR. However, knockdown of circadian clock genes or stimulation of REV-ERBs reveal distinct contributions to cell death in BD patient NPCs, some of which distinguish Li-R and Li-NR.

## Introduction

Bipolar disorder (BD) is a debilitating psychiatric disorder characterized by episodic depression and mania, with disrupted rhythms in sleep and activity [1, 2]. Twin studies indicate that risk for BD is 70–80% heritable and genome-wide association studies (GWAS) have identified > 60 genetic loci with enriched expression in cortical and hippocampal neurons [3, 4]. Brain imaging studies of BD have revealed widespread gray matter volume loss [5, 6]. Post-mortem brain studies in BD suggest neuronal loss involves the retraction of dendrites, and apoptosis-associated signaling pathways [7, 8]. Lithium is an effective drug treatment for BD and attenuates brain gray matter loss [6], but only ~ 30% of BD patients are clinically responsive. There are no reliable ways to predict lithium responsiveness. In animals, brain activation of anti-apoptosis proteins like BCL2 has been implicated in the protective mechanism of lithium [9, 10], but in humans, the molecular mechanisms underlying lithium’s actions remain incompletely described, but may also involve neuroprotection. Lithium-responsiveness may have distinct genetic and neurobiological features that could be leveraged to optimize treatment [11, 12]. Better understanding of lithium’s mechanism of action may therefore be clinically salient.

Circadian rhythm disruption, especially low amplitude rhythms and phase delays are associated with BD [1], and previous work has identified circadian rhythm abnormalities in BD patient cells [13–16]. Cellular circadian rhythms are maintained by well-characterized, transcriptional/translational negative feedback loops comprised of “clock genes”. In this network, BMAL1-CLOCK protein complexes activate rhythmic transcription of *PER1/2/3* and *CRY1/2*. Upon translation, PER and CRY proteins repress BMAL1-CLOCK over recurring cycles of ~ 24 h that sustain cellular circadian rhythms [17]. BMAL1-CLOCK drives expression of additional clock genes encoding REV-ERBα/β and ROR α/β/γ [18]. These proteins co-regulate circadian rhythms and a large number of clock-controlled genes that regulate the rhythmic expression of cellular processes. As examples, BCL2 and TP53 rhythms [19, 20], oscillations in endogenous metabolites such as heme [21] and cholesterol-based lipids [22] may be important mechanisms in temporally regulating proliferation and apoptosis.

Lithium inhibits glycogen synthase kinase 3 (GSK3B) [23], and alters circadian rhythms by modifying the actions of GSK3B on clock proteins, including BMAL1 [24], PER2 [25] and REV-ERBα [26]. Phosphorylation of REV-ERBα by GSK3B increases stability, whereas inhibition of GSK3B destabilizes the protein increases turnover [26]. Therefore, clock proteins including REV-ERBα may be relevant therapeutic targets of lithium. Previous work has determined that lithium response is associated with genetic variation in *REV-ERBα* (also called *NR1D1*) [27, 28]. Small molecules have been developed that target nuclear receptors, including REV-ERB (α/β) and ROR (α/β/γ) proteins [29, 30]. REV-ERB ligands have anti-inflammatory properties [31], but are not well studied with respect to direct effects on circadian rhythms in neurons, or as potential neuroprotective agents in BD.

In addition to being disease markers of BD, circadian rhythms disruptions have been proposed as a means of distinguishing lithium-responders (Li-R) and lithium non-responders (Li-NR) [14, 16, 32]. In cellular models using patient fibroblasts or induced pluripotent stem cell (iPSC)-derived neurons, we have shown that circadian disruption is associated with non-response to lithium [14, 16]. Linking to other research on neuronal loss in BD, our recent work in human fibroblasts has extended this finding to implicate overlap between circadian clocks and apoptosis pathways as potentially important [33]. These studies revealed differences in apoptosis and cellular protection from lithium in BD patient cells compared to controls, that molecular pathways regulating circadian rhythms and apoptosis overlap and that gene expression underlying circadian-apoptosis networks were distinctly organized in healthy controls, and in BD patients, with additional differences between lithium responders (Li-R) and non-responders (Li-NR). In particular, we found that *Per1* knockdown increased rhythm amplitude and was neuroprotective, whereas *Per3* knockdown decreased rhythm amplitude and sensitized cells to apoptosis, indicating regulatory roles of clock genes on apoptosis and potential links between cell death and rhythm amplitude. Similar mechanistic studies of the circadian clock in cell death regulation have not yet been conducted in neurons or in human neuronal models of BD.

Presently, we hypothesized that overlap between neuronal circadian rhythm and cell death pathways would differ in NPCs from control and BD donors, and associate with lithium response. Therefore, we developed models of apoptosis and lithium neuroprotection in mouse neuron and BD patient NPCs. Using these, we examined apoptosis, select components of the circadian clock, and the neuroprotective effects of REV-ERB/ROR nuclear receptor ligands in BD (including Li-R and Li-NR) and control NPC.

## Methods

### Determination of Lithium Response.

Skin biopsies were obtained from controls, or patients with BD type I who participated in the Pharmacogenomics of Bipolar Disorder (PGBD) lithium monotherapy trial [34]. Lithium response was determined by prospective clinical evaluation. Age and sex matched controls were evaluated using The Structured Clinical Interview for DSM-IVTR. Control participants with any psychiatric illnesses were excluded. Most participants were of European ancestry and most BD patients were on medication at the time of biopsy. Characteristics of the cell line donors are shown Tables S1. All research was approved by the VA San Diego Research Committee.

### Immortalized Mouse Hippocampal Neurons.

Immortalized embryonic mouse hippocampal neurons (IMHN, line mHippoE-18) were commercially sourced (Cedarlane). IMHN have a glutamatergic phenotype, express MAP2 synaptic markers, NMDA receptors, AMPA receptors subunits and BDNF. These cellular properties and culture methods have been described previously [35]. To assess circadian rhythms, IMHN were transduced with the *Per2*-luc reporter using lentivirus [13].

### Neuronal Progenitor Lines.

Colonies of iPSCs were grown from BD and control fibroblasts as reported previously [16, 36]. Neural rosettes were expanded in neural proliferation medium [NIM plus 20 ng/ml fibroblast growth factor 2 (Preprotech). Rosettes were dispersed in accutase to form NPCs and passaged every 5–7 d. NPCs were frozen at −160°C until use.

### Circadian Rhythm Assays.

Circadian rhythm experiments were performed in a luminometer, sampling photoemissions every 10 min [13]. Circadian rhythm parameters were estimated by fitting raw data to a damped sine wave using commercial software (Lumicycle Analysis).

### Drugs.

GSK4112, SR8278, SR1001, SR1078, lithium chloride and staurosporine (STS) were purchased from Tocris. Drugs were dissolved in DMSO or water and stored as concentrated solutions at −80°C prior to use.

### Gene Knockdown.

To maximize knockdown, siRNA pools that bind multiple sites were used (SMARTpool siGENOME siRNA, Dharmacon): for REV-ERBα: *NR1D1* (M-003411-02-0005), for BMAL1: *ARNTL* (M-010262-00-0005), *PER1* (M-011350-00-0005), and non-targeting negative control (D-001206-14-05). NPCs were plated in Matrigel-coated 24 well plates at 20,000 cells/well and grown for 48 h. Afterwards, siRNA and DharmaFECT2 Transfection Reagent (Dharmacon, T-2002-02) were mixed with medium and incubated for 20 min at 20°C. This mixture was added to the cells and incubated for 48 h at 37°C following the manufacturer’s protocol.

### Caspase and Viability Assays.

Cells were plated on 96 well-plates (9,000/well) and treated with STS (62.5nM). STS remained in the media with or without any additional drugs for 6–20 h. Using enzymatic bioluminescence assays, Caspase-Glo (Promega G8091) or Celltiter-Glo Luminescent Viability (Promega, G7570), photoemissions were measured with a plate reader (Biotek Cytation 3). Modifications were made for viability assays employing siRNA. Gene knockdown was conducted in 24 well-plates (20,000 cells/well) for 48 h. After STS, 500 μl of viability assay reagent was added. The plate was gently shaken for 2 min and incubated for 10 min at 25°C. Aliquots were transferred to a 96 well plate for assay.

### Gene Expression Analyses.

RNA was prepared by RNeasy kit (Qiagen). cDNA (750 ng) was prepared using reverse transcription (Applied Biosystems). Gene expression was estimated by quantitative real-time PCR using a CFX384 thermocycler (Bio-Rad). Validated Taqman primers (Thermo Fisher Scientific) were used to measure expression of target genes. Expression of each gene was normalized to a non-rhythmic housekeeping gene, *GAPDH*. Target gene expression was estimated by calculating 2ΔCt where ΔCt is the difference in cycle threshold between *GAPDH* and the target gene. Data were normalized to the vehicle treatment of each cell line. All experiments were run in technical triplicates.

## Results

### Cell death in mouse neurons and NPCs.

**Figure 1:**
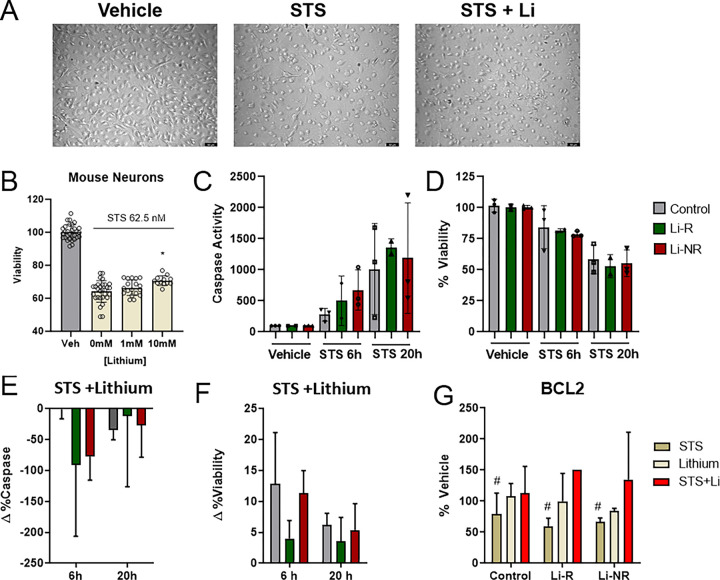
Effects of lithium on staurosporine-induced apoptosis in mouse neurons and human NPCs from BD patients and controls. A) Representative bright field microscopy data of control NPCs following 20 h exposure to 62.5 nM Staurosporine (STS) or vehicle (left panel). Images show significant cell loss from STS (middle panel) and partial reversal (right panel) by co-treatment with STS + lithium (1 mM). B) STS caused decreases in IMHN viability (yellow) compared to vehicle controls (gray) that were attenuated by lithium in a concentration dependent manner, with significant differences in viability STS + lithium 10 mM vs STS alone. C) STS increased in caspase activity in NPC at 6 h and 20 h (with significant effects of drug, time and drug × time interaction, all p<0.01 by two-way ANOVA). There were no significant group differences among control (gray), lithium responder (Li-R, green) and lithium non-responder (Li-NR, red) NPC. D) STS decreased NPC viability at 6 h and 20 h (with significant effects of drug, time and drug × time interaction, all p<0.001 by two-way ANOVA). There were no significant group differences among control (gray), lithium responder (Li-R, green) and lithium non-responder (Li-NR, red) NPC. Lithium (1 mM) given concurrently with STS had neuroprotective effects on NPCs. E) nominally reducing caspase activation (2-way ANOVA was not significant) and F) significantly increasing viability compared to vehicle-treated NPCs exposed to STS (significant effect of lithium, p<0.001 at both 6 h and 20 h). For the effects of lithium on both caspase activity and viability, there were no significant group differences. Data for each donor (n=3 control, 2 Li-R, 3 Li-NR) are expressed as the mean of all technical replicates for each individual donor. F) *BCL2*expression was measured in NPCs from Control, Li-R and Li-NR. STS significantly reduced *BCL2* expression (main effect of drug, p<0.05 two-way ANOVA, indicated by #). Co-treatment of NPCs with lithium + STS resulted in BCL2expression similar to vehicle controls. There was no significant difference in *BCL2*expression across groups or no group × drug interaction. Error bars indicate the standard error of the mean (SEM).

To establish models of neuronal cell death and lithium neuroprotection, we tested the effects of STS on NPCs and IMHNs. After confirming NPC identity using markers of neuronal lineage (Figure S1), microscopic examination of control NPCs indicated that STS reduced cell count and that lithium generally attenuated STS-induced cell loss (Fig. 1A). We then conducted additional biochemical experiments to quantify cell loss and neuroprotection. In IMHN, STS treatment for 20 h caused quantifiable cell death that was significantly attenuated by co-treatment with lithium in a concentration-dependent manner (Fig. 1B). We next used STS to induce caspase activation and apoptosis in NPCs from BD patient and controls. After a brief 6 h exposure to STS, caspase activation in NPCs (N = 3 control, 2 Li-R, 3 Li-NR), increased by approximately five-fold and viability decreased by approximately 20% (Fig. 1C–D). After a longer 20 h STS exposure, caspase activity increased further by approximately ten-fold, and viability decreased further to approximately 50% compared to control levels (Fig. 1C–D). At both times, co-treatment of NPCs with lithium at a therapeutically relevant concentration (1 mM) was neuroprotective. At 6 h after STS, despite having relatively little effect on caspase activity, lithium increased viability, with 90% of NPCs remaining viable (Fig. 1E–F). At 20 h, lithium significantly mitigated this cell loss and improved viability by an average of approximately 5% compared to vehicle co-treatment (Fig. 1E–F). For both caspase activity and viability, there were main effects of lithium treatment and time, but there were no significant group differences between control and BD, or Li-R/Li-NR (Fig. 1C–F). To further characterize neuronal mechanisms underlying apoptosis in BD and control cells, we examined *BCL2* expression, a putative mediator of lithium neuroprotection [37]. Consistent with past reports, *BCL2* expression was decreased by STS and this effect was uniformly reversed by lithium. There were no significant group differences between Control, Li-R and Li-NR NPCs (Fig. 1G). We conclude from these results that both IMHN and human iPSC-derived NPC recapitulate key aspects of lithium’s neuroprotective effects *in vitro* using the STS apoptosis model.

### Clock gene knockdown and viability.

**Figure 2:**
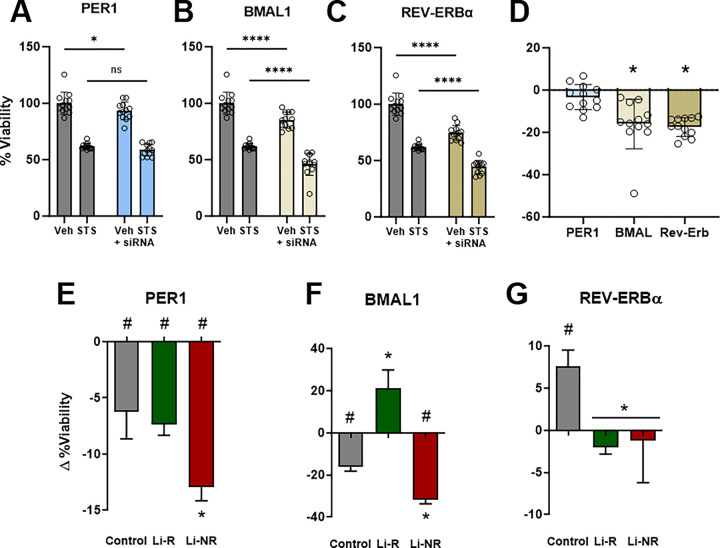
Effects of circadian clock gene knockdown on NPC viability. A) In immortalized mouse hippocampal neurons (IMHN) knockdown of *Per1*(blue), *BMAL1* (white) and *Rev-Erbα*(tan) led to decreased viability in the absence of any other intervention. B) Following treatment with staurosporine (STS), knockdown of *Per1*, *BMAL1*, *Rev-Erbα*further reduced viability. Data for each gene were analyzed using 2-way ANOVA. Results for *Per1*, *BMAL1* indicate additive effects of STS and gene knockdown (groups effects significant, p<0.05). For *Rev-Erbα*, there was a significant STS × siRNA interaction suggesting the effects of knockdown on viability were significantly attenuated under STS-treated conditions. Effects of clock gene knockdown on viability in BD patient (Li-R/Li-NR) and control NPCs treated with STS. There were no group differences in responses of NPCs to STS alone. Compared to STS alone C) *PER1* siRNA significantly decreased NPC viability across all groups, but viability was significantly greater in Li-NR compared to controls and Li-R. D) *BMAL1* siRNA significantly decreased viability of control and Li-NR, but increased viability of Li-R. E) *REV-ERBα* knockdown increased the viability of control NPCs, but significantly decreased the viability of BD (Li-R and Li-NR) NPCs.

To determine the contributions of individual clock genes to apoptosis, we next knocked down expression of *BMAL1*, *PER1* and *REV-ERBα* in IMHN prior to STS. For each gene, knockdown caused specific changes in the cell death responses to STS. After *PER1* knockdown in IMHN, viability was modestly decreased compared to controls at baseline, but knockdown did not affect the response to STS (Fig. 2A). In contrast, knockdown of *BMAL1* and *REV-ERBα* both significantly reduced viability in vehicle- and STS-treated IMHN (Fig. 2B–C). Knockdown of both *BMAL1* and *REV-ERBα* independently reduced viability, and following STS, knockdown additively increased cell death (Fig. 2B, 2D). Following *REV-ERBα* knockdown, there was an additional significant siRNA × STS interaction, whereby *REV-ERBα* knockdown modestly attenuated the amount of cell death caused by STS: −17% viability for REV-ERBα vs. −25% for sham (Fig. 2C, 2D). Having established these findings in IMHNs, we next conducted similar experiments in control and BD patient NPCs, including Li-R and Li-NR. Compared to control siRNA, *PER1* knockdown increased cell death, in both vehicle- and STS-treated NPCs. However, the amount of additional cell death differed significantly by group (Fig. 2E). Viability decreased the most in NPCs from Li-NR (−12.9%) compared to Li-R (−7.4%) and controls (−6.2%). Following *BMAL1* knockdown (Fig. 2F), viability was decreased − 16.1% in controls. In Li-NR, viability was significantly lower (−31.9%), while in Li-R NPCs, *BMAL1* knockdown increased viability (+ 21.2%). REV-ERBα knockdown caused a modest increase in viability in control NPCs after STS (+ 7.6%) but as in IMHN, caused a nominal decrease in viability in the two BD groups (−1.2% to −2.0%). The overall effect of REV-ERBα knockdown was significantly different between control and BD samples, but not between Li-R and Li-NR (Fig. 2G). These results reveal differences in the role of clock genes regulating cell death in BD patient NPCs that associate with lithium response.

### Pharmacological modulators of the circadian clock.

**Figure 3:**
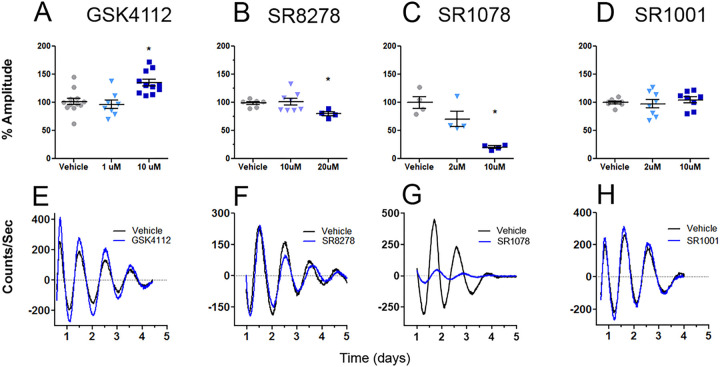
Small molecule ligands targeting REV-ERBs and RORs have distinct effects on circadian rhythms. Using IMHN expressing the Per2-luc reporter, circadian rhythms were evaluated for effects on amplitude using four distinct drug treatments that each target nuclear receptors involved in regulating circadian rhythms in different ways. A) The REV-ERB agonist GSK4112 showed concentration-dependent effects on rhythms with no significant effect at 1 μM, but a significant increase in amplitude at 10 μM. B) The REV-ERB antagonist showed no significant effect at 10 μM, but a significant decrease in amplitude at 20 μM. C) The ROR agonist SR1078 showed concentration-dependent effects on rhythms with no significant effect at 2 μM, but a significant amplitude decrease at 10 μM. D) The ROR inverse agonist SR1001 had no significant effect on rhythms at either of the concentrations tested (2 μM and 10 μM). All analyses were conducted using one-way ANOVA with post-tests to identify differences between groups (n= 4–12/group). Statistical significance was determined using p<0.05 indicated by *. Data shown in E-H are representative traces of vehicle (black) and drug-treated (blue) IMHNs, observed over a 5-day recording period. To emphasize the rhythmic component of the light emission signal, data were baseline subtracted using a detrended running average.

Given the indications that there may be distinct contributions to cell survival from clock genes in NPC from BD patients, we next used pharmacological modulators of the circadian clock to further investigate these interactions. Both in IMHN and NPC, we first tested lithium, a drug that has effects on REV-ERBα [26]. In cellular rhythm assays, lithium increased amplitude in IMHN, similar to its effects on rhythms in other cells [13] (Figure S1A). In NPCs, lithium increased amplitude in control, but not Li-R or Li-NR samples (Figure S1B-D). We next tested four drugs that target the REV-ERB and ROR nuclear receptors. Compared to NPC, IMHN are better suited to studies using multiple drug conditions in parallel and were the focus of these experiments. The REV-ERB agonist GSK4112 also increased rhythm amplitude, whereas the REV-ERB antagonist SR8278 decreased amplitude. The ROR agonist SR1078 decreased amplitude, whereas the ROR inverse-agonist SR1001 had no effect on rhythms (Fig. 3). Since GSK4112 and lithium both increased circadian rhythm amplitude, we hypothesized that these shared amplitude-increasing effects may be relevant for neuroprotection and focused on GSK4112 in subsequent studies.

### Neuroprotective properties of a REV-ERB agonist.

**Figure 4:**
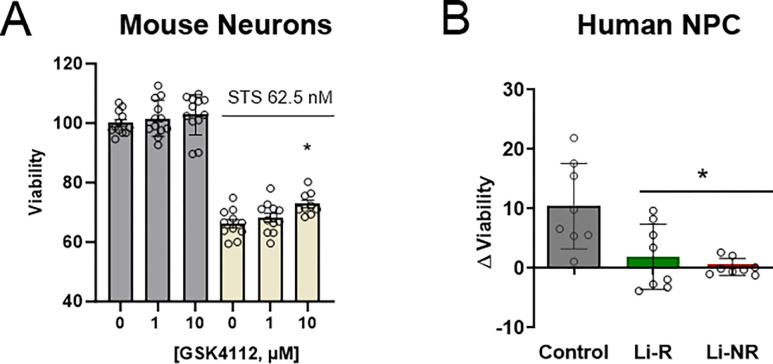
REV-ERB agonists promote cell survival in mouse neurons and control NPC, but not in NPCs from BD patients. A) To assess IMHN, neurons were treated with the REV-ERB agonist GSK4112 (0, 1 or 10 μM) at the same time as STS (62.5 nM) or vehicle. Survival assays were conducted 18 h after STS treatment. Compared to IMHN receiving STS and no additional treatment, GSK4112 caused a concentration-dependent increase in cell survival following STS with a significant increase observed in the 10 μM GSK4112 group (p<0.05 indicated by *). Analysis was conducted using one-way ANOVA with post-tests to identify differences between groups (n= 9–12/group). B) To assess NPC from control and BD Li-R/Li-NR donors, viability experiments were conducted in a similar manner using STS and GSK4112 at 10 μM concentration. To normalize for minor baseline differences, data are expressed as the difference in viability following STS treatment alone and STS+GSK4112 co-treated samples (higher number indicates greater protective effect of GSK4112). Co-treatment of control NPC with GSK4112 caused a significant increase in viability of 10.5 ±2.5% (mean ± SEM), whereas there was no significant effect on viability in the NPCs from Li-R (1.9 ± 1.9%) or Li-NR (0.2 ± 0.5%). NPC experiments were performed with 8 technical replicates/group, from n=2 control, n=2 Li-R and N=1 Li-NR donors. In post-tests, the group difference between controls and BD (combined Li-R and Li-NR) was statistically significant (indicated by * p<0.05).

Knockdown of *REV-ERBα* altered cell death responses in IMHN and NPC. Moreover, the REV-ERB agonist GSK4112 and lithium both increase amplitude, perhaps indicating neuroprotection may be related to the strength of circadian rhythms. Therefore, we hypothesized that a REV-ERB agonist may be neuroprotective. To test this, we conducted viability experiments with GSK4112 both in IMHN and NPCs. Compared STS exposure alone, co-treatment with GSK4112 and STS increased IMHN viability in a concentration dependent manner that was statistically significant at 10 μM (Fig. 4). In NPCs from controls, GSK4112 (10 μM) had a similar protective effect, significantly increasing viability after STS exposure by 10%, similar to the effects observed in IMHN. However, in NPCs from either BD group (Li-R and Li-NR), GSK4112 had no effect on viability, leading to a statistically significant group difference between control and BD in the protective benefit from the drug (Fig. 4, p < 0.005).

### Mechanisms of REV-ERB agonist neuroprotection in NPCs.

**Figure 5:**
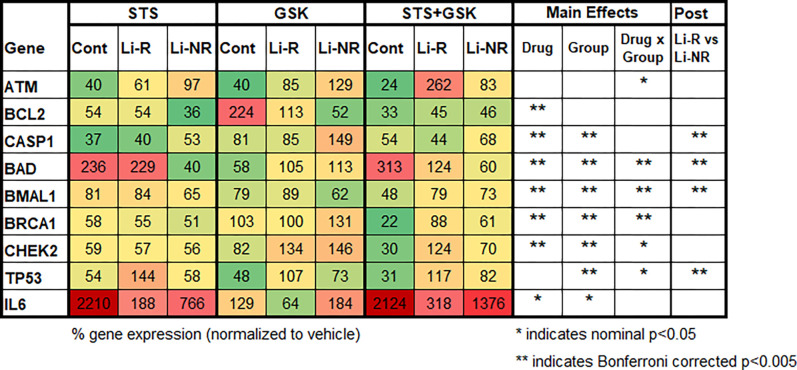
Gene expression profiles following STS and GSK4112 in BD patient and control NPCs. To investigate potential mechanisms underlying differential response to the neuroprotective effects of GSK4112, nine candidate genes with roles in apoptosis or neuroprotection were examined. Identical cultures of NPCs were treated in parallel with vehicle, STS, GSK4112 or STS+GSK4112 for 6 h before sample collection and analysis to identify upstream changes preceding cell death. Normalized data are shown as % of vehicle control. Results are color coded as indicated. Green: strong downregulation; Yellow: modest downregulation; Orange: modest upregulation; Red: strong upregulation. Data were analyzed using a 2-way ANOVA with factors of group and drug. In cases where ANOVA revealed a significant group difference, post-hoc T-tests were conducted between Li-R and Li-NR. To control for multiple comparisons of 9 genes and 2 factors, a Bonferroni correction was applied to the data. After Bonferroni correction, statistical significance was defined as p<0.005 (indicated by **). Nominally significant findings (p<0.05) that did not survive correction are also shown (indicated by *). To reduce batch effects, each experiment used the same cDNA sample and was run as technical triplicates for each gene from n=2 control, n=2 Li-R and N=1 Li-NR donors.

The differences in neuroprotective effects of GSK4112 in control and BD NPC were evaluated further to identify potential underlying mechanisms. In NPCs from control and BD (Li-R and Li-NR), we compared the effects of STS and GSK4112 (alone and in combination) on the expression of nine genes selected for their involvement in cell death and neuroprotection (Fig. 5). Most of the genes selected (7/9) showed significant differences in expression after drug treatments, indicating that the drug interventions effectively engaged the intended target genes. Following drug treatment, *BAD*, *BCL2*, *BMAL1*, *BRCA1*, *CASP1*, *CHEK2* and *IL6* showed significantly altered expression (all p < 0.05 in 2-way ANOVA, main effect of drug). An overlapping, but distinct set of genes (7/9) showed significant group differences and differed among control, Li-R and/or Li-NR samples: *BAD*, *BMAL1*, *BRCA1*, *CASP1*, *CHEK2*, *IL6* and *TP53*. *ATM* expression did not differ by group (trend p = 0.05) or drug alone, but revealed a significant drug × group interaction. Based on the findings that GSK4112 increased viability after STS treatment in control, but not in BD samples, we looked for gene expression patterns that correlated with these viability findings. Following treatment with STS, *IL6* expression was massively upregulated in NPCs from controls, but significantly less so in BD cells (Control 2210% vs 188% Li-R and 766% in Li-NR). Following combined treatment with STS + GSK4112, *BAD* expression directly correlated with the viability results, with significantly increased levels in control NPC, but not in either BD group (Control 313%, Li-R 124% and Li-NR 60%). Other gene expression patterns inversely correlated with that viability effects using the same drugs. *BMAL1*, *BRCA1*, *CHEK2* and *TP53* were all downregulated by combination treatment with STS + GSK4112 in controls, but upregulated by STS + GSK4112 in BD NPCs (Li-R and Li-NR, Fig. 5). Interestingly, other effects on gene expression of combined treatment of STS + GSK4112 did not correlate well with viability following STS + GSK4112, but showed significant differences between Li-R and Li-NR, and distinguished the BD sub-groups: *CASP1*, *BAD*, *BMAL1* and *TP53*.

## Discussion

We investigated the overlap between circadian rhythms and neuroprotection in mouse and human neuronal cells to identify potential biological mechanisms underlying disease susceptibility to BD and model lithium response *in vitro*. Our work shows that STS-induced apoptosis is mitigated by lithium across mouse and human neuronal models, including in control and BD-patient derived NPCs, supporting the use of these models for mechanistic studies of lithium’s neuroprotective actions. We show for the first time in neuronal cells the distinct contributions of individual clock genes in regulating cell death. In NPCs, reduced clock gene expression reveals differences in cell death responses between BD and controls (*REV-ERBα*), and between BD sub-groups, Li-R and Li-NR (*PER1* and *BMAL1*). Additional differences in cell death were revealed by the neuroprotective response to REV-ERB nuclear receptor ligands in BD patient and control NPCs, differences that correlate with gene expression in cell death associated pathways. By using BD patient NPCs from well-characterized Li-R and Li-NR, we can link our findings with relevant clinical endpoints, thereby increasing the potential future translational impact of the work. In the broader context of circadian rhythm disruption in BD, these results indicate that one of the consequences of circadian disruption may be disruption of regulatory pathways involved promoting neuronal survival. Based on our results, we conclude that disruptions across overlapping networks of circadian clock and cell survival genes may be a factor contributing to BD and/or affect the subsequent treatment response to lithium. However, the interventions tested in this study both to induce cell death and disrupt rhythms were non-physiological. It is not yet clear how the differences we have identified are expressed in the brain in vivo, and under what environmental circumstances they may be most relevant.

Our previous work revealed that BD and control fibroblasts differ in the anti-apoptosis effects of lithium, but that the pro-survival benefit of lithium did not distinguish Li-R and Li-NR [33]. Our current study also found that there is no major difference in the cell death response to STS, or protective response to lithium that distinguishes Li-R and Li-NR. This may relate to the potency of STS as an inducer of cell death that may overwhelm the neuroprotective benefit of lithium and mask group differences in beneficial effects. However, in both our fibroblast and NPC studies, we did observe differences in BD sub-groups among individual clock genes. In particular, our work in neuronal cells indicate that *PER1*, *BMAL1*, and *REV-ERBα* have regulatory rolesin cell death that are broadly conserved across mouse and human neuronal models, but with species-specific and disease-specific differences. Genomic studies reveal genetic overlap of BD with chronotype, sleep and circadian entrainment [4,38]. Therefore, some of these neuronal cell mechanisms may overlap with pathways conferring risk for BD and/or responsiveness to lithium. *Per1* knockdown led to modest increases in cell death in IMHN, with larger effects on NPCs. *PER1* knockdown in BD and control NPCs increased the sensitivity of cells to apoptosis and caused greater reductions in viability, particularly in Li-NR NPCs which were most sensitive to the intervention. *PER1* is an immediate early gene that couples the cellular circadian clock to environmental signals [39]. Accordingly, we speculate that PER1 may be involved in some of the intracellular responses to STS that regulate stress-response pathways that determine cell survival or death, e.g. through mediators such as P53. In our previous fibroblasts study, we found significant correlation of *PER1* expression with the cell survival gene *TP53* in control cells that was disrupted in BD [33]. Previous studies have documents functional interactions between PER1 and P53 proteins [40], and in studies of BD patients combining brain imaging and gene expression suggest that *TP53* may contribute to the neuroprotective effects of lithium [41]. P53 is likely only one important node in this neuronal network. Additional studies are required to define the role of PER1 and its other signaling partners.

We conclude based on current and past evidence, that *BMAL1* and *REV-ERBα* also interact with celldeath pathways, perhaps as negative regulators. In the canonical clock network, *BMAL1* is essential forclock function, andREV-ERB proteins are *BMAL1* repressors. Loss of *REV-ERBα* in control NPCs increased cell survival but had no effect on viability in BD cells in either Li-R and Li-NR. Knockdown of *BMAL1* revealed additional group differences between Li-R and Li-NR, increasing viability in Li-R and reducing it in Li-NR. In our previous fibroblast study, we found disrupted co-expression of *BMAL1* and *BRCA1* in BD, and differences in *REV-ERBα* and *BRCA1* co-expression that distinguished Li-R and Li-NR [33]. *BRCA1* can regulate apoptosis, and in a case-report, a cancer causing a *BRCA1* mutationwas linked to lithium-responsive BD with circadian disruption [42,43]. To study the link between *BMAL1* and REV-ERB further, we used drugs that act upon the ROR and REV-ERB transcription factors and alter circadian rhythms. We selected GSK4112 to study in survival assays since it shared the amplitude increasing properties of lithium. Previous studies have revealed that similar REV-ERB agonists are protective in models of neuroinflammation [31]. We found that GKS4112, a REV-ERB agonist that amplifies the *Per2*-luc circadian rhythm has neuroprotective properties that are comparable to lithium. Interestingly, both lithium and GSK4112 have features that distinguish control and BD NPCs: Similar to BD patient iPSC-neurons [16] and fibroblasts [13,14], lithium increases rhythm amplitude in control NPCs, but not BD cells. Nonetheless, lithium is equally neuroprotective across groups. Conversely, the neuroprotective effects GSK4112 are observed in control, but not BD NPCs. Paradoxically, the neuroprotection pattern of GSK4112 was similar to *REV-ERBα* knockdown, implying that any engagement of REV-ERBα, either stimulation or repression may reduce apoptosis under some circumstances, perhaps implicating temporal dynamics over expression level as the primary mechanism of REV-ERBα-mediated neuroprotection.

Gene expression changes following STS alone and STS ± GSK4112 provide plausible candidate mechanisms underlying the differing neuroprotective responses in BD and control NPCs. For example, following STS treatment of NPCs, *IL6* expression was significantly higher in control compared to BD patient NPCs, suggesting attenuated *IL6* induction by STS. Responses to STS+GSK4112 treatment also differed significantly in BD and control NPCs. For instance, after STS+GSK4112 treatment, *BMAL*1 expression decreased in controls but increased in BD NPCs. Suggesting co-regulation among genes, *BRCA1* expression showed the same pattern, consistent with neuroprotection from GSK4112 in controls and lack of effect of in BD NPCs. Compared to controls, BD NPCs also downregulated *BAD* expression, and upregulated *CHEK2* and *TP53*. *BMAL1*, *TP53*, *BAD* and *CASP1* expression also distinguished Li-R and Li-NR. We hypothesize that these observed responses to GSK4112 reflect complex drug actions across the network of rhythmic, clock-controlled genes that regulate cell survival pathways, and that lack of effect in BD indicates disruption of this network [16]. However, we cannot rule out other explanations, including GSK4112 effects on non-circadian targets.

### Limitations.

Our work has some important limitations. Mouse neurons and NPCs may have properties *in vitro* that differ importantly from mature neurons *in vivo*. Our approach was limited by having cell lines from a small number of individuals and may have been underpowered to detect small effects. The results of many of our experiments are complicated by rhythmic and oscillating gene expression. In our studies, drugs were not administered to target at any particular time in the circadian cycle. Therefore, our findings could indicate differences in network signaling, or phase differences in expression rhythms. With only a single time point to evaluate, these possibilities cannot be disentangled. We conclude that REV-ERB nuclear receptor modulators of the circadian clock may have important functions as experimental tools to identify points of convergence in circadian-cell survival networks in cellular models of BD, but face limitations as neuroprotective agents given the lack of effects in BD NPCs.

## Conclusions

This study advances our understanding of the molecular mechanisms underlying apoptotic cell death in BD and its potential links to circadian rhythm disruption. These findings may have relevance to gray matter loss in the brains of BD patients, and the neuroprotective effects of lithium. Moreover, the work highlights the potential for circadian rhythms, including circadian clock proteins as a therapeutic intervention for BD [44]. Further research is warranted to validate these findings, define mechanisms in more detail and determine which aspects may be amenable for translation into clinically relevant treatments for BD patients, particularly those who do not respond to lithium therapy.
